# 2459. Prevalence of Nasopharyngeal Carriage of Macrolide and Lincosamide Resistance Genes Among a Household-based cohort of Children and Adults in a Peri-urban Community in Peru 

**DOI:** 10.1093/ofid/ofad500.2077

**Published:** 2023-11-27

**Authors:** Cara Charnogusrky, Ana Gil, Lucie Ecker, Rubelio Cornejo, Stefano Rios, Mayra Rosana Ochoa, Bia M Peña Peralta, Omar Flores, Claudio Lanata, Carlos G Grijalva, Leigh M Howard

**Affiliations:** Vanderbilt University Medical Center, Nashville, Tennessee; Instituto de Investigacion Nutricional, Lima, Peru, Lima, Peru; Instituto de Investigación Nutricional, Lima, Lima, Peru; Instituto de Investigacion Nutricional, Lima, Peru, Lima, Peru; Instituto de Investigacion Nutricional, Lima, Peru, Lima, Peru; Instituto de Investigación Nutricional, Lima, Lima, Peru; Instituto de Investigación Nutricional, Lima, Lima, Peru; Instituto de Investigación Nutricional, Lima, Lima, Peru; Instituto de Investigacion Nutricional, Lima, Peru, Lima, Peru; Vanderbilt University Medical Center, Nashville, Tennessee; Vanderbilt University Medical Center, Nashville, Tennessee

## Abstract

**Background:**

Antibiotic resistant infections commonly occur in healthcare settings, but the prevalence of antibiotic resistant genes (ARGs) in healthy individuals in the community is unknown. We provide an initial assessment of nasopharyngeal carriage of ARGs conferring resistance to macrolides and/or lincosamides, including erm, mph, ABC-F, and lnu genes.

**Methods:**

Nasopharyngeal swabs were systematically obtained at enrollment and weekly thereafter from children and adults enrolled in a household-based prospective cohort study in Lima, Peru. Samples were sequenced using the Illumina Respiratory Pathogen/ID AMR Panel to detect common respiratory bacteria and ARGs. We defined ‘any erm gene’ (erm) as the detection of any specific erm gene class, ‘any mph gene’ as detection of any specific mph gene class, ‘any ABC-F gene’ as any specific ABC-F gene class, and ‘any lnu gene’ as any specific lnu gene detection. We compared the prevalence of erm, mph, ABC-F and lnu gene colonization at enrollment among age groups (ages 0-4, 5-17, 18-44, and 45+ years) using the Fisher's exact test. We then compared the frequency of co-detection of two or more macrolide or lincosamide ARGs between both pediatric age groups (ages 0-17 years) and both adult age groups (18+ years).

**Results:**

114 individuals were included in this analysis (Table 1). At least one macrolide or lincosamide ARG was detected in 61/114 (53.5%) individuals. The prevalence of erm, mph, ABC-F, or lnu gene detection was similar among age groups (Figure 1). Two or more genes were co-detected in 45/114 (39.4%) individuals. Co-detections with two or more erm, mph, ABC-F, or lnu genes were more frequent in children 0-17 years compared to adults 18+ years (p=0.013) (Figure 2).

**Figure:**
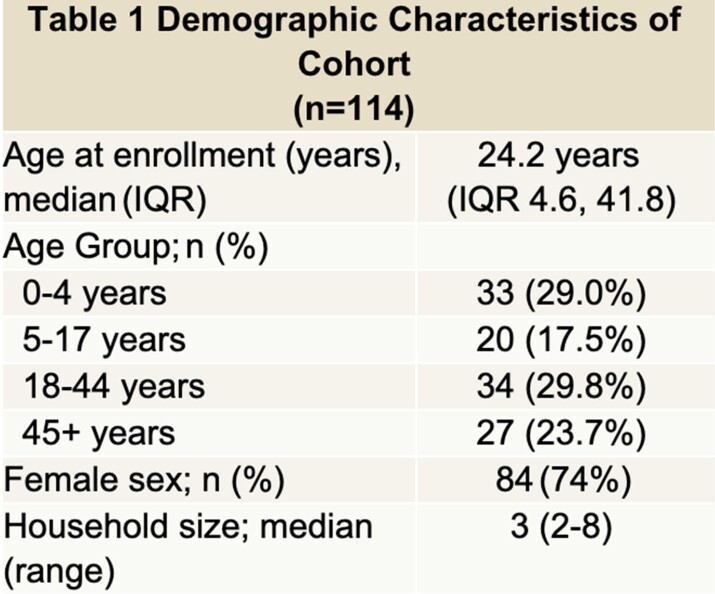

**Figure:**
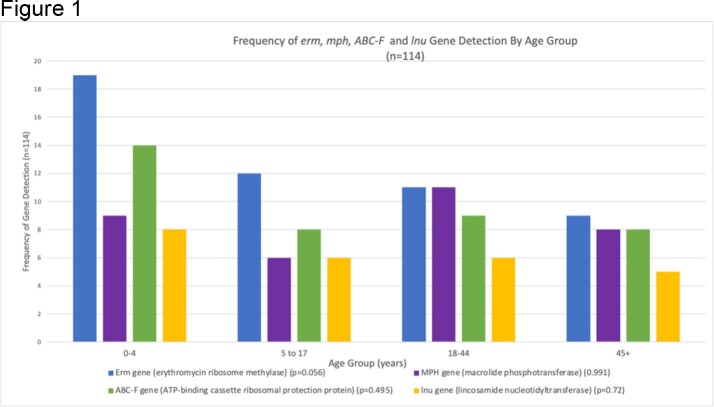

**Figure:**
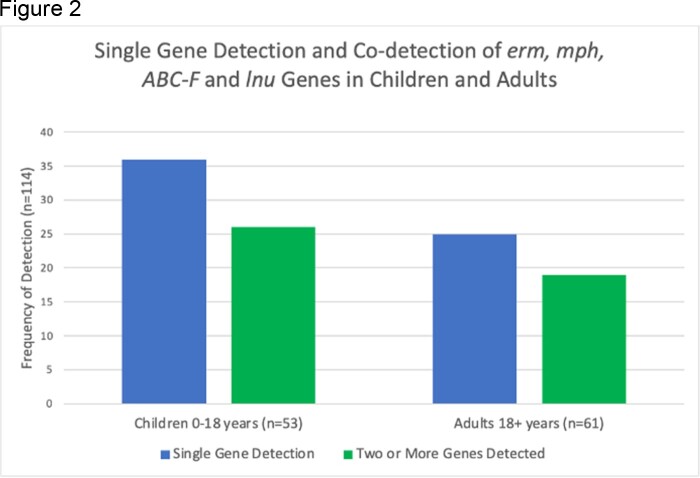

**Conclusion:**

Conclusions: These preliminary results indicate that macrolide or lincosamide ARGs were commonly detected in healthy community-dwelling children and adults in Lima, Peru. The prevalence of erm, mph, ABC-F, and lnu genes was similar across age groups, and co-detection of ARGs was more common in children. Future studies will assess changes in ARG carriage over time, transmission among household members, and its clinical relevance.

**Disclosures:**

**Claudio Lanata, MD, MPH**, CureVAc AG Germany: Grant/Research Support|HilleVac Inc.: Advisor/Consultant|HilleVac Inc.: Grant/Research Support **Carlos G. Grijalva, MD, MPH**, AHRQ: Grant/Research Support|CDC: Grant/Research Support|FDA: Grant/Research Support|Merck: Advisor/Consultant|NIH: Grant/Research Support|Syneos Health: Grant/Research Support

